# Great Achievements under Suffering

**Published:** 1893-08-12

**Authors:** 


### Great Achievements under Suffering.

### II.—THE BLIND POET.

Not Milton. To him blindness came late in life after years of travel and minute observation had stored his memory with images of beauty. The poet of whom we would speak now lost his sight almost wholly at the age of three years, and could never have had one clear recollection of the outside world.

Philip Bourke Marston, born August 13th, 1850, was a direct descendant of John Marston, the Elizabethan dramatist. His father was himself a poet and dramatist, and his god-parents, Philip Bailey, author of "Festus," and Mrs. Craik, seemed to supply additional links to the literary career which was early his choice. Some indistinct glimmers of light visited him during some years after the accident which deprived him of sight in babyhood, but even these ceased at a later period and left his darkness unrelieved. He seems to have had no systematic education such as is open now to the blind. Towards the close of his life he learned to use a typewriter, but as a boy he learned neither to read nor write, But as a listener from early years he was a lover of books, and the daily home life supplied an education in itself. Among his father's friends were Browning, Thackeray, Dickens, Rossetti, Swinburne, and many others noted n literature and art. From such intercourse, and the intimate knowledge of their works which followed in natural course, he graduated in a true poet's university. It might be judged that no reading would be so acceptable to the blind boy as that which could carry him out of the darkness in which he sat into a world of of light and colour. From admiration to imitation is an easy step, and thus it came about that his poetic inspiration early found a vent in descriptions of nature. presenting a record of secondhand impressions curiously modified by a sensitive brain. The influence of Rossetti and Swinburne, the latter predominantly, may be traced in all his poems.

The details of his life, as given by Miss Louise Moulton in the memoir prefixed to the new edition of the poems, are beyond measure sad. During his boyhood it was to his mother he owed all the sympathy and devoted attendance of which he stood peculiarly in need. She was his amanuensis and confidante, on whom his deepest affections centred. When he was scarcely twenty she died, exposing him for the first time to a sorrow and desolation the more overwhelming that from the nature of his affliction he was debarred from the active work which at such times is the only healthy resource. He felt then "as if the whole world had gone to pieces." It was but the first of a series of such blows. A year later his engagement with Miss Nesbit seemed to mark the beginning of a fresh period of hope and achievement. His first volume of poems, "Song-tide," was published and kindly received, and for a few months no cloud threatened the new happiness which had visited his shaded life. Then within the year, after a brief period of agonizing suspense, Miss Nesbit died, carried off by swift consumption, and the darkness closed in once more, literally as well as figuratively, for after the time of intense grief which followed, his eyes no longer retained even the slightest sensitiveness to light.

From this time his sister Cicely devoted herself to him entirely. "It seemed almost as if his heart beat, his brain throbbed in her body, so entirely was it the business and the pleasure of her life to do his will." The two lived together in London, breaking long studious months by travels in France and Italy, and surrounded by many friends. One friend in special, Oliver Madox Brown, a rising artist and writer, fell under the sway of Marston's personal influence. The two formed a romantic friendship, and became inseparable. It was one of the supreme joys of Marston's life, but after two years of the closest intimacy he again found himself bereaved. In 1874 his friend died of blood poisoning, after a short illness, which had supplied no preparation for the news of his death Marston's second volume of poems, "All in All," was then in the press, and the haunting pain which makes each line a separate sting seems almost a presage of the new sorrow preparing. Readers will differ in their estimate of these sonnets to his dead love. Some will always reckon their unbearable pathos as a high triumph of skill. To others it will seem that they lack the breadth of outlook and strong handling essential to the translation of personal sorrow into art.

Cicely was now his only comfort and chief friend, and her devotion knew no bounds. But it seemed all through his life as if death followed swift after those on whom his love rested. Only four years after his friend's death his sister, too, was called away from him. She died very suddenly of apoplexy during the absence of her brother in France, leaving his life desolate and bereaved indeed. "When his mother, his betrothed, and his friend died in sad and swift succession, there had always been Cicely to comfort and console him. But when Cicely went, there was no such survivor." Many new friends came into his life; many sorrows were still to be lived through. But life had done its best and its worst for him before he reached his thirtieth year. He was still to witness the death of his father, of his surviving sister Eleanor, of his much-loved brother-in-law Arthur O'Shaughnessy, and of friend after friend, before he was summoned in his turn. He died February 14th, 1887.

> Who now from far above, with eyes alight
> And spirit enkindled, haply towards us here
> Looks down, unforgetful yet, of days like night,
> And love that has yet his sightless face in sight.

So Swinburne wrote. And many others of those whom he had delighted to honour lent their tribute to his work and memory.

A sad and great life. Unspeakably sad in the sorrows which marked indeed, as his biographer points out, its chief events. Great too, in its sorrows, from the spirit of high endurance with which they were encountered, and from the glad humility with which he welcomed the little daily joys, never lacking to those who will stoop to gather them. "To the very last," says Miss Moulton, "he could be blithe whenever there was anyone with whom to be merry, and small things sufficed to please and cheer him." A great and sad life, too, in its promise and fulfilment, for his songs were almost wholly of love and of nature, and death warred with his love as relentlessly as blindness with his perception of the beautiful. His love poems are nearly all of the nature of requiems, and need to be read in the light of his life's story. His tears scald as they fall, and the restless pain finds no stay of consolation.

But by the seashore, or out among the woods, and in his garden, Nature yielded him her choicest secrets. What are colour and form to the thousand tokens by which beauty reveals her presence to the soul of the blind man? That which we lightly pass over, absorbed in the more obvious beauties of Nature, was all in all to him. Every tone of the wind voices, their touch on his hair or cheek, the dash of the "bright spray" in his eyes, the feel of the rain on his shoulder, "soft, subtle scent of sweet flowers blossoming," "the sea-scent, warm and sharp and sweet," the change of air on the cheek from sun to shade, the fall of the least leaf, the movement of each delicate petal among the roses and lilies he loved so well—these are some of the secrets he learnt to understand in his garden. Touches such as these makes his garden fancies unique among descriptive poems, for a supersensitiveness to scent and sound and touch seem not only to atone for the lost sense, but to supply it. We cannot believe but that he saw as in a vision the living things he loved, whose inmost souls he learned to read. To the reader his garden is an enchanted spot, full of spirit voices and presences invisible but very real. There is passion and pain here, too, but dew of comfort upon all:—

Hope of summer, love of Roses
Certainty that sorrow closes.

The hope flickers and burns dim, but never wholly dies:—

If life be full of comfort, fair and sweet,
I will be meekly thankful that my feet
Are spared the stones that wound, and as I may
Try to make smooth for others, a rougher way;
But should life bitter prove, and incomplete,
This pain of living it is very fleet,
And rest will come, with quiet set of day.

